# Chemopreventive Role of Apigenin against the Synergistic Carcinogenesis of Human Papillomavirus and 4-(Methylnitrosamino)-1-(3-pyridyl)-1-butanone

**DOI:** 10.3390/biomedicines8110472

**Published:** 2020-11-04

**Authors:** Fangzheng Yin, Lijiao Zhao, Lili Zhang, Yuhe Chen, Guohui Sun, Jintao Li, Na Zhang, Yuancong Xu, Paul Kay-Sheung Chan, Rugang Zhong

**Affiliations:** 1 Beijing Key Laboratory of Environmental and Viral Oncology, Faculty of Environment and Life, Beijing University of Technology, Beijing 100124, China; yinfangzheng2017@emails.bjut.edu.cn (F.Y.); zhanglily2017@emails.bjut.edu.cn (L.Z.); chenyh@emails.bjut.edu.cn (Y.C.); sunguohui@bjut.edu.cn (G.S.); ljt2008@bjut.edu.cn (J.L.); nanatonglei@bjut.edu.cn (N.Z.); xuyuancong@bjut.edu.cn (Y.X.); lifesci@bjut.edu.cn (R.Z.); 2 Departments of Microbiology, Faculty of Medicine, The Chinese University of Hong Kong, Shatin, Hong Kong; paulkschan@cuhk.edu.hk

**Keywords:** tobacco smoke, human papillomavirus, apigenin, 4-(methylnitrosamino)-1-(3-pyridyl)-1-butanone, synergistic carcinogenesis, chemoprevention

## Abstract

Tobacco smoke and human papillomavirus (HPV) are both crucial causes of cancer, and their cooperative carcinogenesis has drawn more attention in recent years. Apigenin (AP), a typical flavonoid abundantly found in flowers of plants, vegetables, and fruits, has been demonstrated to exert an anti-carcinogenic effect on various types of cancer. In this study, we investigated the capability of AP against malignant transformation and DNA damage of immortalized human esophageal epithelial (SHEE) cells induced by the synergism of HPV18 and 4-(methylnitrosamino)-1-(3-pyridyl)-1-butanone (NNK). The results indicated that the enhancement of migration, invasion, and proliferation ability of SHEE cells induced by HPV and NNK could be effectively inhibited by AP. Moreover, the levels of pyridyloxybutylated (POB)-DNA adducts induced by NNK via P450-catalyzed metabolic activation could also be significantly suppressed by AP. Further analyses on the molecular mechanism revealed that AP inhibited the synergistic carcinogenesis of NNK and HPV on SHEE cells by reducing the expression of mutp53, CDK4, Cyclin D1, and p-Rb (Ser 780), increasing caspase-3 activity, thereby arresting the cell cycle at G1 phase and promoting apoptosis of SHEE cells. We hypothesize that the decrease in NNK-induced POB-DNA adduct levels is related to the deactivation of P450 by AP, which needs to be confirmed in future studies. This study highlights that AP may be employed as a promising chemopreventive agent against cancers in smokers with an HPV infection.

## 1. Introduction

Tobacco smoke is an important cause of cancer, contributing to 22% of all cancer deaths and 71% of lung cancer deaths worldwide [1]. Tobacco-specific nitrosamines (TSNA) are a class of carcinogenic substances, in which 4-(methylnitrosamino)-1-(3-pyridyl)-1-butanone (NNK) is considered to be highly harmful to humans and is classified as a Class I carcinogen by the International Agency for Research on Cancer (IARC) [2]. The carcinogenic mechanism of NNK is mainly involved in the metabolic activation catalyzed by cytochrome P450, followed by induction of DNA adducts. Hecht’s group provided convincing evidence that NNK results in the formation of pyridyloxybutylated (POB)-DNA adducts after P450-catalysed hydroxylation [3,4] and established a robust quantitation method of total POB-DNA adducts by determining the amount of (4-hydroxy-1-(3-pyridyl)-1-butanone) (HPB) released from these adducts [5]. Besides DNA damage induction, the carcinogenesis of NNK also involved in protein regulation in cell cycle and apoptosis, such as cyclin D1, Bcl-2, and Bax, leading to cell proliferation or anti-apoptosis [6,7].

Viral infection is another important cause of cancer, which can cooperate with chemical carcinogens in the development of cancer. High-risk human papillomavirus (hr-HPV) is well recognized as a cause of malignant tumors in cervical, vulvar, vaginal, penile, and nongenital regions, such as lung, bronchus, and oropharynx [8,9,10,11,12,13]. hr-HPV encodes E6 and E7 oncoproteins, which induce p53 degradation by binding with p53 protein and affects cell transformation by binding to tumor suppressor proteins, respectively [14,15]. In recent decades, a number of epidemiological evidences indicate that the synergistic effect between tobacco smoke and hr-HPV shows associations with the occurrence and development of cervical cancer, as well as head and neck cancers [16,17,18,19,20]. Our recent investigation reported that NNK and HPV-18 synergistically promote malignant transformation and DNA damage of human esophageal epithelial cells (SHEE) [21]. Therefore, it is necessary to elucidate the mechanism and explore possible chemoprevention of the synergism between NNK and hr-HPV.

Apigenin (4′,5,7,-trihydroxyflavone, AP), a flavonoid phytochemical abundantly existing in vegetables and fruits, plays an important role in the chemoprevention of malignant tumors. The anti-tumor activity of AP is mainly in the aspects of inhibiting angiogenesis, preventing the malignant transformation of cells, reducing cell invasion and metastasis, regulating cell signal transduction pathways, promoting apoptosis, and modulating cell cycle [22,23,24,25,26,27]. Several studies demonstrated that AP has a good inhibitory effect on cancers induced by tobacco smoke or hr-HPV infection. AP significantly inhibited HPV-transformed human prostate epithelial cells by modulating cell cycle and apoptotic machinery [28]. The malignant proliferation of human pancreatic cancer cells induced by NNK can be prevented by AP by inhibiting the activation of NNK via focal adhesion kinase (FAK) and extracellular regulated protein kinases (ERK) signaling pathways [29]. Moreover, AP selectively inhibited HPV-positive Hela and SiHa cells but showed decreased toxic effects on HPV-negative C33A and HaCaT cells [30]. These evidences suggest that AP is a potential chemopreventive agent for cancers induced by NNK and HPV. In this study, we investigated the chemopreventive effect of AP on the inhibition of the synergism between NNK and HPV, which will assist in the prevention and treatment of cancers related to smoking and HPV infection.

## 2. Experimental Section

### 2.1. Chemicals and Reagents

AP was purchased from Tokyo Chemical Industry (Tokyo, Japan). NNK, HPB, and [3,3,4,4-D4]4-hydroxy-1-(3-pyridyl)-1-butanone ([D_4_]HPB) were obtained from Toronto Research Chemicals (North York, ON, Canada). All other chemicals, reagents, and solvents were acquired from Sigma-Aldrich (St. Louis, MO, USA).

### 2.2. Cell Culture

Immortalized human esophageal epithelial cells (SHEE) were purchased from the BeNa Culture Collection (BNCC) Biotechnology Company (Beijing, China) and were transfected with the HPV-18 E6E7 gene (SHEE-E6E7) or with empty vector (SHEE-V) by lentiviral transfection described in our previous study [21]. Cells were cultured using Dulbecco’s Modified Eagle Medium (DMEM, Hyclone, South Logan, UT, USA) supplemented with 10% fetal bovine serum (FBS, DAKEWE, Shenzhen, China) and 1% penicillin and streptomycin (Hyclone, South Logan, UT, USA).

### 2.3. Wound Healing Assay

The literal migration of cells was measured using wound-healing assays. SHEE cells were pretreated with AP for 24 h accompanied by an AP-free group as controls. Wounds were artificially created in the confluent cellular monolayer by scraping with a 10-μL pipette tip followed by washing the remaining adherent cells three times with PBS. Then the remainder of the adherent cells were exposed to 10 μM NNK in a serum-free medium for 48 h. The images of the wounds were taken at different time intervals using an inverted microscope (Olympus IX51, Tokyo, Japan), and the cell migration rate was calculated by the following formula:

Migration rate = (*S* − *S*_0_)/(1 − *S*_0_) × 100%

Here, *S* and *S*_0_ referred to the percentages of the areas covered by cells in the total area at 48 and 0 h, respectively.

### 2.4. Transwell Assay

Cell invasion was examined using transwell migration chambers (8 μm pore size, Corning, NY, USA). SHEE cells were pretreated with AP for 24 h followed by exposure to 10 μM NNK for 24 h, accompanied by a control group without the addition of NNK or AP in the medium. Then, after being cultured in a serum-free medium for 12 h, the cells were inoculated in the upper chamber covered by Matrigel (BD Biosciences, Franklin Lakes, NJ, USA) for 48 h, while the lower compartments were filled with 600 μL of DMEM with 20% FBS as a chemoattractant. After being stained with 0.1% crystal violet solution (Beyotime, Beijing, China), the migratory cells were counted under an inverted microscope at 200-fold magnification.

### 2.5. Cell Colony Formation Assay

SHEE cells seeded in 6-well plates at a density of 250 cells/mL were pretreatment with AP for 24 h followed by exposure to NNK for another 24 h. After a 7-day incubation, colonies were stained with 0.1% crystal violet for photography. The number of colonies containing more than 50 cells was counted. The colony-forming efficiency (CFE) was calculated as (number of colonies/number of cell inoculations) × 100%.

### 2.6. Cell Apoptosis and Caspase-3 Activity Assay

The apoptosis of SHEE cells was analyzed by dual staining with Annexin V-FITC/propidium iodide (PI) (Dojindo, Kumamoto, Japan). Cells were pretreated with AP for 24 h and then exposed to NNK for 24 h. Then the harvested cells were resuspended in a binding buffer followed by the addition of Annexin V-FITC and PI and were analyzed by a FACSCalibur flow cytometer (BD Biosciences, San Jose, CA, USA). Caspase-3 activity was evaluated using a kit according to the manufacturer’s instructions (Beyotime, Beijing, China). The absorbance at 405 nm of each sample was measured by a Multiskan FC plate reader (Thermo Fisher Scientific, Waltham, MA, USA).

### 2.7. Hochest Staining

The nuclei of apoptotic cells were stained using Hoechst 33342 (Solarbio Science & Technology Co. Ltd., Beijing, China). Briefly, 3 × 10^4^ cells were plated in each well of a 6-well plate and grown at 37 °C for 24 h. After treatment, cells were stained by Hoechst 33342 for 30 min in an ice bath followed by washing three times with PBS. Then images of the cells were recorded by an IX-51 inverted fluorescence microscope (Olympus Corporation, Tokyo, Japan).

### 2.8. Cell Cycle Analysis

Cells cycle was analyzed using flow cytometry. Briefly, the cells exposed to AP for 24 h, followed by 24-h NNK treatment were harvested and washed with PBS, then fixed with 70% ethanol containing 1% FBS at 4 °C overnight. After an additional washing step, 500 μL of staining buffer containing 10 μL of RNase A (50×) and 25 μL of PI (20×) was added to each sample. After incubation at 37 °C for 30 min in the dark, the samples were analyzed by a FACSCalibur flow cytometer (BD Biosciences, San Jose, CA, USA).

### 2.9. Western Blot

Treated cells were collected and resuspended in lysis buffer with the addition of protease and phosphatase inhibitors (Beyotime, Beijing, China). Total protein was quantified using a BCA quantification kit (Beyotime, Beijing, China). Then the protein samples denatured at 95 °C for 5 min were separated by 10% sodium dodecyl sulfate polyacrylamide gel electrophoresis (SDS-PAGE) and transferred to polyvinylidene fluoride (PVDF) membranes. After blocking with 5% skimmed milk at room temperature for 2 h, the membranes were washed and incubated with primary antibodies, including anti-p53, anti-mutant p53, anti-Rb, anti-CDK4, anti-Cyclin D1, anti-β-actin, anti-GAPDH (Boster Biological Technology, Pleasanton, CA, USA) at 1:400 dilution and anti-p-Rb (Ser780) (Cell Signaling Technology, Danvers, MA, USA) at 1:1000 dilution overnight at 4 °C. After washing, the membranes were incubated with secondary antibodies for 1 h. Image acquisition and data analysis were performed using an Odyssey CLx Imager and Image Studio software (LICOR Biosciences, Lincoln, NE, USA).

### 2.10. HPLC-ESI-MS/MS Quantitation of HPB

After pretreated with AP for 24 h, SHEE cells were exposed to NNK in a freshly prepared medium containing 1% S9 rat liver microsome for another 24 h. Then the collected cells were subjected to DNA isolation as described in our previous study [21]. The obtained DNA was dissolved in deionized water containing 30 nM [D_4_] HPB as the internal standard, followed by acidic hydrolysis with HCl at 80 °C for 3 h to release HPB. The samples were purified by solid-phase extraction (SPE) using 30-mg 1 cm^3^ Strata X cartridges (Phenomenex, Torrance, CA, USA) followed by centrifugal vacuum freeze-drying (Savant SpeedVac, Thermo Electron, San Jose, CA, USA). Subsequently, the samples were re-dissolved in water and analyzed by high performance liquid chromatography-electrospray ionization tandem mass spectrometry (HPLC-ESI-MS/MS, TSQ Quantum Discovery Max, Thermo Electron, San Jose, CA, USA). HPLC separation was performed on a 150 mm × 2.0 mm Luna C18 (2) 5 μm column (Phenomenex, Torrance, CA, USA). The gradient of the mobile phase started from 35% to 90% methanol in 15 mM ammonium acetate over a period of 20 min, followed by an isocratic elution for 3 min. Then it returned to 35% methanol in 2 min, followed by an equilibration for 13 min, at a flow rate of 0.1 mL/min. The determined levels of HPB-releasing DNA adducts were calculated as per our previous study and expressed as fmol per mg DNA [31,32].

### 2.11. Statistical Analysis

The results were expressed as mean ± standard deviation (SD). Values between groups were compared using Student’s *t*-test. A *p*-value of less than 0.05 was considered statistically significant.

## 3. Results

### 3.1. AP Inhibited Literal Migration Ability of SHEE Cells

**Figure 1:**
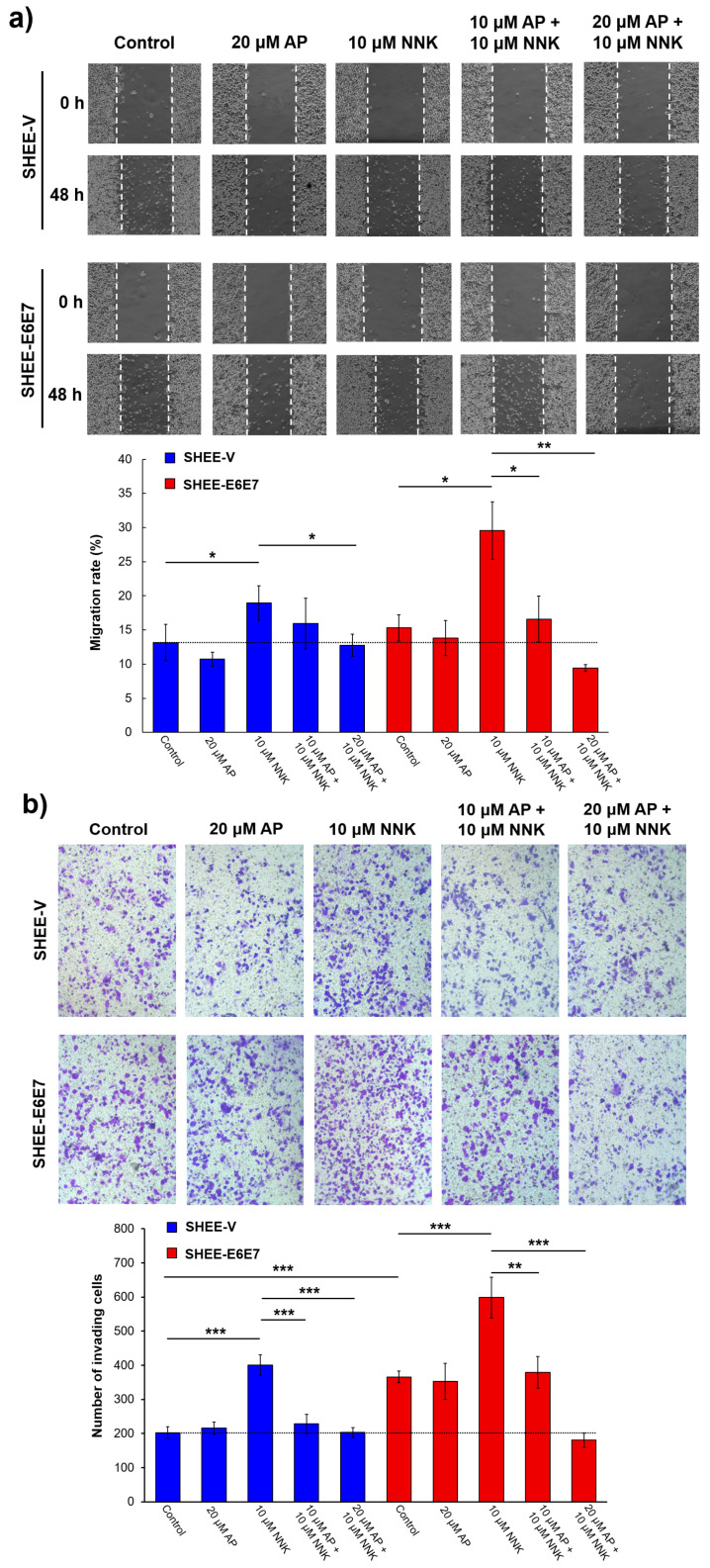

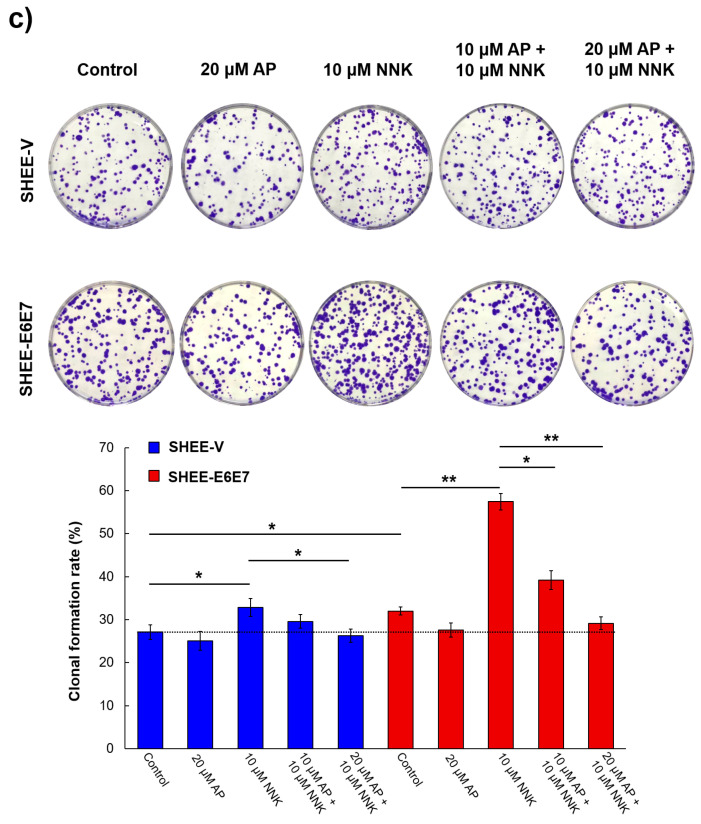
Apigenin (AP) inhibited the increased migration, invasion, and proliferation ability induced by the synergism of 4-(methylnitrosamino)-1-(3-pyridyl)-1-butanone (NNK) and human papillomavirus (HPV). (**a**) wound healing ability of immortalized human esophageal epithelial (SHEE) cells after 48-h NNK exposure with AP pretreatment (× 100); (**b**) cell invading capacity of SHEE cells after 24-h NNK exposure with AP pretreatment (× 200); (**c**) cell colony formation of SHEE cells after 24-h NNK exposure with AP pretreatment followed by a 7-day incubation. Data are presented as mean ± SD (*n* = 3). * *p* < 0.05, ** *p* < 0.01, *** *p* < 0.001.

The literal migration of SHEE-E6E7 and SHEE-V cells treated by NNK and NNK + AP was monitored by cell scratch assays. As shown in Figure 1a, AP had no obvious influence on the migration of both cell lines, while NNK resulted in a significant enhancement of the migration rate (*p* < 0.05) with the highest level observed in SHEE-E6E7 cells (29.57 ± 4.19%). However, for cells pretreated with AP, the migration ability of both cell lines was inhibited compared with the non-AP exposed, NNK-treated groups. In particular, in the groups of 20 μM AP, the migration rates of SHEE-V (12.76 ± 1.63%) and SHEE-E6E7 (9.43 ± 0.52%) markedly decreased to levels similar to those of the SHEE-V control group (13.15 ± 2.67%). These results demonstrate that AP effectively inhibits the synergism between HPV and NNK in SHEE cells’ migration.

### 3.2. AP Suppressed Cell Invasion Promoted by NNK and HPV

The metastatic ability of SHEE cells after various treatment was determined using a transwell invasion assay. Similar to the results of migration assay, AP had no significant effect on the invasion ability of SHEE cells, while a remarkable elevation was observed in the groups treated by NNK (*p* < 0.001, Figure 1b). The number of invading cells in the NNK-treated SHEE-E6E7 group was 1.49 and 1.63 times that of SHEE-V treated by NNK (*p* < 0.05) and SHEE-E6E7 without NNK treatment (*p* < 0.05), respectively, indicating that NNK and HPV synergistically promote the invasion ability of SHEE cells. Meanwhile, a statistically significant difference was observed between NNK-treated cells with and without AP pretreatment. The number of invading cells for SHEE-V and SHEE-E6E7 after AP (20 μM) + NNK exposure decreased significantly to 51% and 30% (*p* < 0.05), respectively, of those treated with NNK alone. The results of cell invasion experiments were in line with the migration assays, indicating that AP can effectively eliminate the promotion of malignant transformation caused by NNK and HPV.

### 3.3. AP Impeded Cell Colony Formation Induced by NNK and HPV

Colony formation assays were performed to verify whether the proliferation of SHEE cells could be impeded by AP. As we expected, AP exhibited potent prophylactic ability against the synergistic effects of HPV and NNK (Figure 1c). The clonal formation rates of NNK-treated SHEE-E6E7 cells with 10 and 20 μM AP exposure prominently reduced by 32% (*p* < 0.05) and 49% (*p* < 0.01), respectively, in comparison with the SHEE-E6E7 cells treated with NNK alone. Taken together, results of the above phenotypic assays show that the malignant transformation induced by the synergism of NNK and HPV can be successfully prevented by AP.

### 3.4. AP Induced G1 Arrest of SHEE Cells

**Figure 2:**
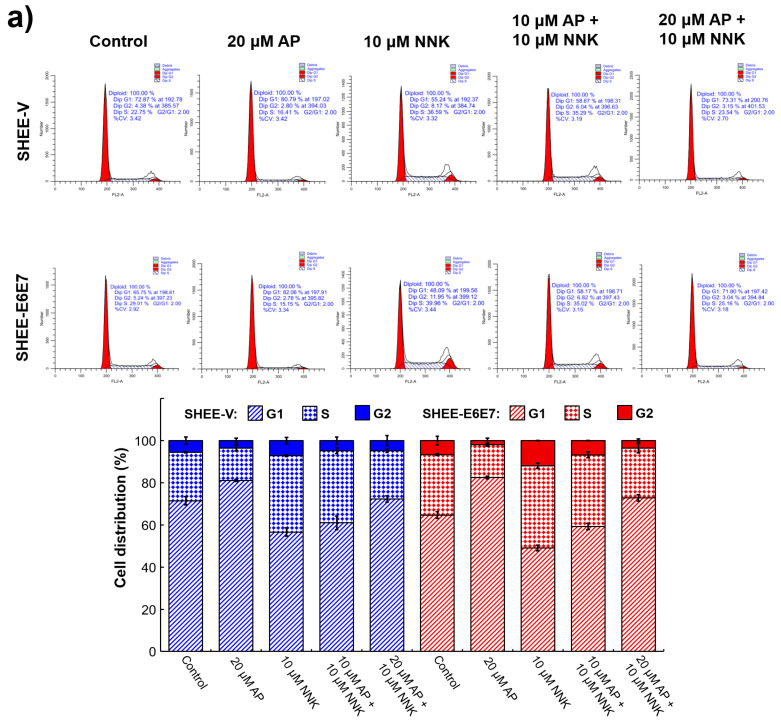

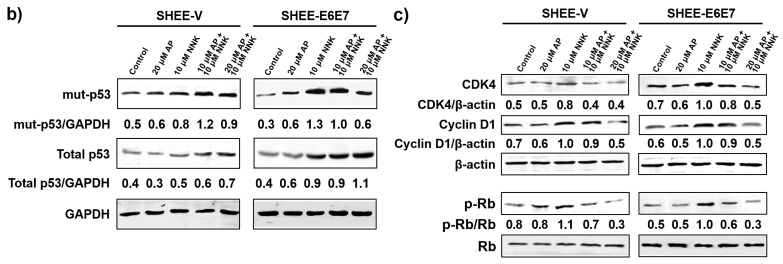
AP prevented cell cycle progression of SHEE cells promoted by the synergism of NNK and HPV and decreased protein expression involved in cell cycle regulation. (**a**) cell cycle distribution of SHEE cells; (**b**) total p53 and mutp53 expressions in SHEE; (**c**) expressions of CDK4, cyclin D1, and p-Rb in SHEE.

As AP was believed to be capable of regulating cell cycle progression, the effect of AP on the cell cycle of NNK-treated SHEE cells and related protein expression were examined to delineate the underlying mechanism. The cell cycle distribution (Figure 2a) indicated that AP pretreatment led to obvious cell cycle arrest in both SHEE-V and SHEE-E6E7 cells. For the groups treated with AP alone, the percentages of the G1 phase in both cell lines (around 80%) increased significantly (*p* < 0.05) compared with their respective control groups (73% for SHEE-V and 66% for SHEE-E6E7), while for the groups treated with NNK alone, the percentages of the G1 phase significantly decreased (*p* < 0.05) to 55% and 48% for SHEE-V and SHEE-E6E7 cells, respectively, accompanied by an obvious increase in the proportion of cells in S and G2 phase. With AP pretreatment before NNK exposure, the percentages of the G1 phase in both cell lines were close to those of control groups, and the proportion of cells in S and G2 phases decreased. Therefore, both SHEE-V and SHEE-E6E7 cell growth inhibition attributed to AP may be caused by G1 phase arrest in vitro. The expression of cell cycle related proteins was analyzed by Western blotting. As shown in Figure 2b, the levels of mutant p53 of both cell lines were increased by NNK exposure but obviously decreased when pretreated by AP in SHEE-E6E7 cells. The expression levels of CDK4, Cyclin D1, and p-Rb (Ser 780) were markedly downregulated in both cell lines pretreated by AP compared with their corresponding groups exposed to NNK only (Figure 2c), which may facilitate SHEE cells to be arrested at the G1/S checkpoint.

### 3.5. AP Promoted Apoptosis of SHEE Cells

**Figure 3:**
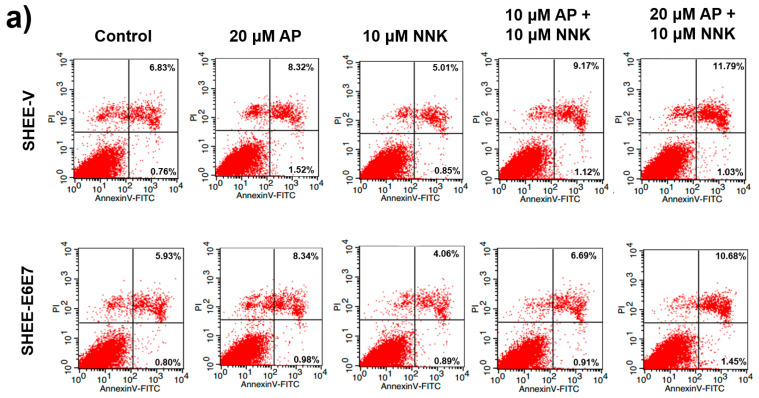

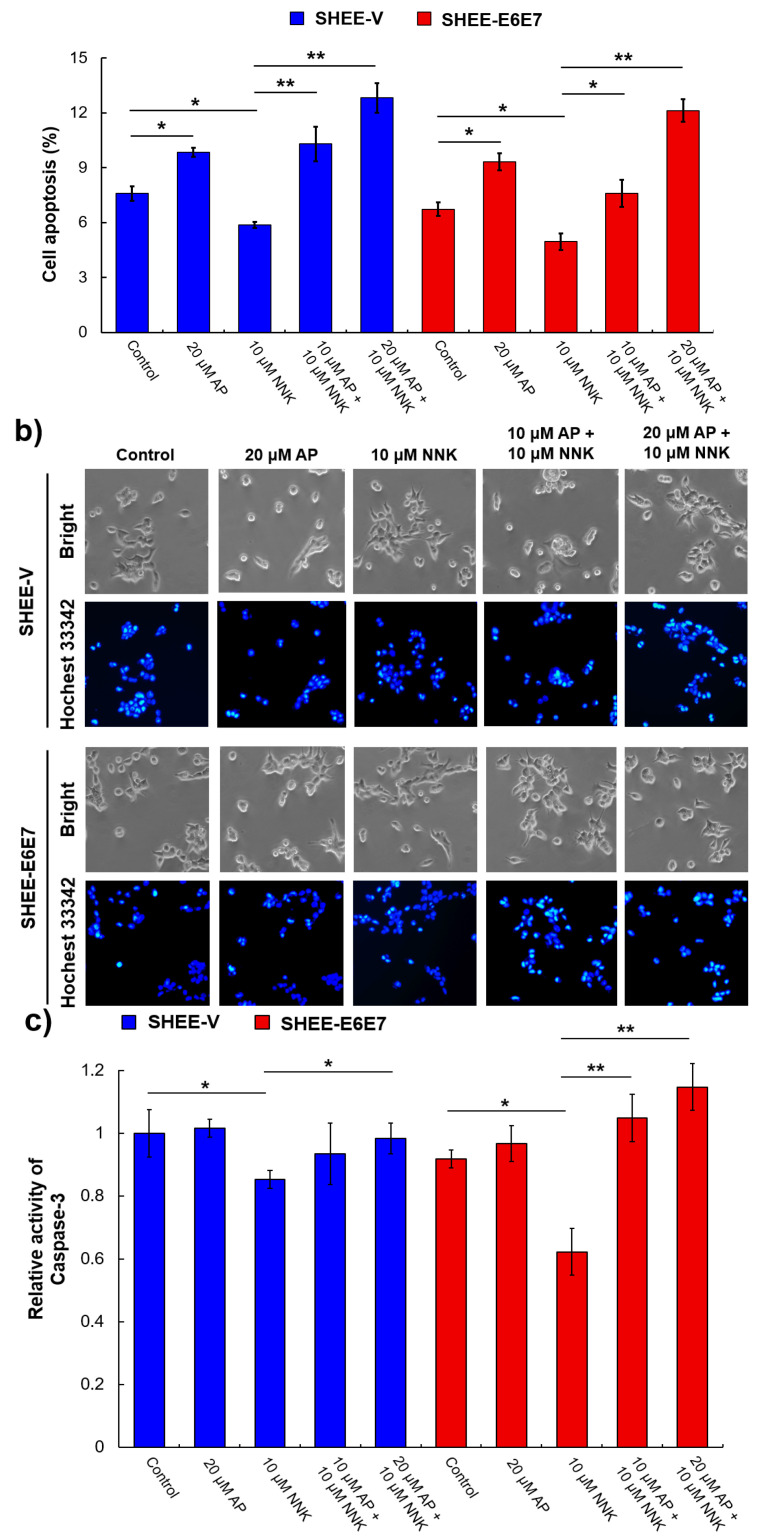
AP prevented anti-apoptosis of SHEE cells promoted by the synergism of NNK and HPV. (**a**) flow cytometry analysis for apoptotic cell number of SHEE; (**b**) photofluorography for validating the apoptotic SHEE cells; (**c**) caspase-3 activity of SHEE cells. Data are presented as mean ± SD (*n* = 3). * *p* < 0.05, ** *p* < 0.01.

Apoptosis of SHEE cells after AP and NNK treatment was measured by flow cytometry. As shown in Figure 3a, NNK prevented apoptosis of SHEE-V and SHEE-E6E7 cells, with the proportion of apoptotic cells reduced to 5.86% and 4.95%, respectively. In contrast, apoptosis of both cell lines was remarkably enhanced with AP pretreatment before NNK exposure. The apoptosis of SHEE cells was inspected by fluorescence microscopy imaging using Hochest 33342. As shown in Figure 3b, AP pretreatments led to more cells exhibiting bright blue fluorescence than those exposed to NNK alone, and morphological changes, such as cell shrinkage and chromatic agglutination, appeared in some of the apoptotic cells. Caspase-3 activity, which is regulated by p53 protein and plays an important role in the process of apoptosis, was measured to evaluate the apoptotic effects of AP. As shown in Figure 3c, the caspase-3 activity in both cell lines was significantly decreased by NNK but was recovered by AP to levels comparable to the control groups. These results together indicate that AP can effectively prevent the anti-apoptotic effect of NNK and HPV and, therefore, inhibit cell proliferation.

### 3.6. AP Protected SHEE Cells from DNA Alkylation Induced by NNK

**Figure 4:**
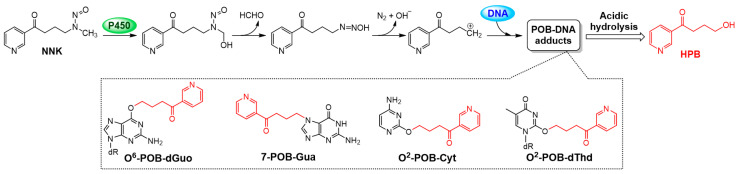
P450-catalyzed metabolic activation of NNK and formation of POB-DNA adducts, mainly including O^6^-POB-dGuo, 7-POB-Gua, O^2^-POB-Cyt, and O^2^-POB-dThd, which release HPB under acidic hydrolysis.

**Figure 5:**
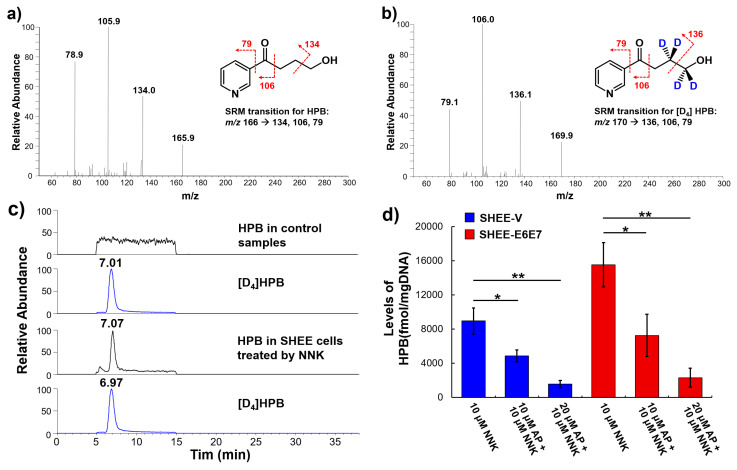
AP suppressed the levels of pyridyloxybutylated (POB)-DNA adducts induced by NNK in cooperation with HPV. (**a**) fragmentation of HPB and spectra of MS2 full scan; (**b**) fragmentation of the isotope-labeled [D_4_]HPB and spectra of MS2 full scan; (**c**) representative selective reaction monitoring (SRM) chromatograph of the DNA samples from the control and NNK-treated groups; (**d**) determined levels of HPB in SHEE cells after 24-h NNK exposure with AP pretreatment. Data are presented as mean ± SD (*n* = 3). * *p* < 0.05, ** *p* < 0.01.

The carcinogenic mechanism of NNK is mainly involved in the formation of POB-DNA adducts induced by metabolic activation of NNK catalyzed by cytochrome P450 (Figure 4). Therefore, HPB, which is released from acidic hydrolysis of POB-DNA adducts, was quantified using HPLC-ESI-MS/MS to determine the total level of POB-DNA adducts in both cell lines after NNK and AP treatment. Selective reaction monitoring (SRM) was performed with the transitions of *m*/*z* 166→79, 106 and 134 for HPB (Figure 5a), and *m*/*z* 170→79, 106 and 136 for the internal standard [D_4_]HPB (Figure 5b). Representative SRM chromatographs obtained from the analysis of controls and test samples are shown in Figure 5c, in which HPB was co-eluted with [D_4_]HPB at around 7 min. No signal was observed in the control samples and the cells exposed to AP alone, indicating that the method had good specificity without contamination in the sample matrix. As shown in Figure 5d, the level of HPB in SHEE-E6E7 cells was significantly higher than that of the SHEE-V group after NNK treatment (*p* < 0.05). A significant decrease (*p* < 0.05) in HPB levels was observed in the groups pretreated by AP compared to those without AP pretreatment. These results demonstrate that AP inhibits the formation of POB-DNA adducts induced by NNK and HPV.

## 4. Discussion

There is increasing epidemiological evidence to suggest synergism between tobacco carcinogens and HPV infection in cancer induction [16,17,18,19,20,33,34]. Furthermore, synergism between tobacco carcinogens and hr-HPV was also observed in in vitro experiments using human cells derived from cervical, lung, and oral cancers [35,36,37,38,39,40,41]. AP, a typical flavonoid with low toxicity and multiple beneficial bioactivities, has been evaluated as a promising candidate for chemoprevention against a variety of cancers. In this study, the ability of AP in blocking the synergism between NNK and HPV was explored by cytophenotypic assays, flow cytometry, Western blot and HPLC-ESI-MS/MS quantitation of NNK-induced POB-DNA adducts. The results demonstrated that AP could effectively prevent the malignant transformation of SHEE cells induced by the synergistic effect of NNK and HPV.

**Figure 6:**
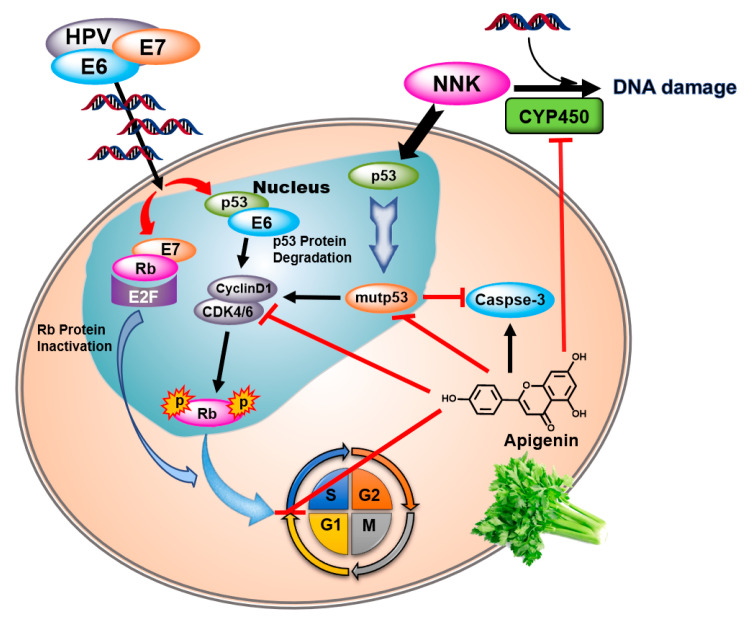
Molecular mechanism of AP inhibiting the synergistic carcinogenesis of HPV and NNK. The arrows and T-bars mean stimulation and inhibition, respectively.

AP exerts chemoprevention via complicated molecular mechanisms, involving blocking cell cycle progression in the subG1 phase and inducing cell apoptosis by regulating the expression of cell cycle related proteins, causing activation of caspase and p53 [42,43]. To elucidate the mechanism of AP in blocking the synergistic carcinogenesis of NNK and HPV, we investigated the changes in the cell cycle, apoptosis, as well as the expression of p53, mutp53, CDK4, cyclin D1, and p-Rb in SHEE cells. A marked increase in mutp53 expression in SHEE-E6E7 cells was observed after NNK exposure, suggesting that NNK and HPV18-E6E7 together promote cancer development by diminishing the tumor-suppressive effect of p53. As depicted in Figure 6, NNK-induced p53 mutations further led to the upregulation of cyclin D1, CDK4, and p-Rb (Ser 780), which resulted in a significant increase in the percentage of cells entering the S phase and a corresponding decrease in the G1 phase. However, AP pretreatment before NNK exposure caused a decrease in the expression of mutp53, Cyclin D1, CDK4, and p-Rb (Ser 780), and the subsequent malignant proliferation of SHEE cells was prevented with G1 phase arrest. As a main terminal cleaving enzyme in the process of apoptosis, caspase-3 was apparently deactivated by the synergistic action of NNK and HPV via inducing mutation of p53, but this action was effectively reversed by AP through reactivation of caspase-3. Although the anti-cancer potential of AP has been examined in numerous studies, the effects of AP on NNK-induced procarcinogenesis has been rarely reported before this study; only Pham et al. [29] observed that AP suppressed the NNK-induced proliferation and migration of pancreatic cancer cell by inhibiting the activation of β-AR and its downstream signals FAK and ERK. Therefore, the chemopreventive mechanism of AP against the synergistic carcinogenesis of HPV and NNK needs to be further explored by examining the signaling pathways but not limited to the PI3K/AKT, MAPK/ERK, JAK/STAT, NF-κB, and Wnt/β-catenin pathways [44].

Since DNA damage plays an important role in cancer occurrence and malignant cellular transformation, NNK-induced DNA damages have been demonstrated in numerous in vivo and in vitro investigations. However, few evidences have been provided for the DNA alkylation by NNK in HPV-infected cells. In the present and our previous studies [21], we consistently observed that the level of total POB-DNA adducts in HPV-positive cells was significantly higher than that in HPV-negative cells, suggesting synergistic carcinogenesis of HPV and NNK. Moktar et al. [45] reported that single- and double-strand DNA breaks induced by cigarette smoke condensate were dose-dependent and persistent in HPV-transformed human ectocervical cells, which gave support to our results. To prevent the synergistic DNA-damaging function of HPV and NNK, we tried to discover a natural product as a potential chemopreventive agent against the synergistic carcinogenesis of HPV and NNK. AP has been proved to protect DNA from various types of damages induced by carcinogens, such as benzo(a)pyrene (BaP)-induced DNA strand breaks [46], ultraviolet-induced cyclobutane pyrimidine dimers [47], and BaP-induced DNA adducts [48]. In the present study, inhibition of NNK-induced POB-DNA adducts by AP was observed for the first time. We presumed that the decrease in POB-DNA adduct levels might be owing to the inhibition of cytochrome P450 (CYP) involving in NNK metabolic activation by AP. This speculation can be supported by the observations of effective inhibition of CYP2A6 and CYP2A13 in vitro by AP [49] and reduction in hepatic CYP2E1 expression in AP-treated mice [50]. Although the underlying mechanism of the chemoprevention of AP is complicated, the inhibition of the synergistic carcinogenesis of HPV and NNK by AP is believed to be closely connected with DNA damages, which warrants further investigation.

## 5. Conclusions

In summary, this study demonstrated that AP inhibits the synergistic carcinogenesis of NNK and HPV on SHEE, which effectively prevents malignant transformation and DNA damage. The possible mechanism of chemoprevention associated with AP could be the arrest of the cell cycle in the G1 phase by downregulating mutp53, CDK4, cyclin D1, and p-Rb, and blocking P450-catalyzed metabolic activation of NNK. This study provides insights into the function of AP, which may be a promising chemopreventive agent against cancers, particularly, in smokers with HPV infection.
